# Curing and Teaching

**Published:** 1923-08

**Authors:** 


THE
### HOSPITAL and HEALTH
REVIEW

### CURING AND TEACHING.

THERE could be no more conclusive proof of the value of the co-ordinating work of the last few years than Sir Napier Burnett's Report for the year 1922 upon the Voluntary Hospitals in Great Britain outside London. This is the fourth of these annual reports, and it is by far the most encouraging. It shows that improvement has been made in almost every direction. Income has increased, expenditure has diminished, interest has deepened, and, above all, it has been made finally clear that the voluntary hospitals can hold their own without the need of State or municipal interference. It is true that, in the aggregate, they have still failed to pay their way, but it has been a brilliant failure. In 1921 the ordinary income of the provincial hospitals fell short of their ordinary expenditure by £419,000; last year the deficit was barely £75,000. For this happy change we have, in the main, to thank the fall in the cost of maintaining institutions which are necessarily costly; yet there has been a very substantial increase in ordinary income—as Sir Arthur Stanley says in the introductory note to the report, "from every quarter help has come." This is conclusive of the hold which the voluntary system has obtained over the country, and we may fairly take it that all classes are united in their determination to maintain the hospitals in their present relation to the people. They exist for the people and will be kept going by the people.

The enormous part which the hospitals play in the national life is graphically illustrated by the fact that during the past year more than two million individual new patients were treated at a cost of five millions of money—this, it will be remembered, is exclusive of London, which is a whole hospital world of itself. Of this huge expenditure not much more than three-quarters of a million comes from interest on invested funds—the other four millions or more are derived entirely from voluntary contributions in one form or another. So long as Imperial and local taxation remain at their present level, it is hopeless to expect a large number of great benefactions—for some years to come, at all events, we must look to the shillings and guineas of the working and middle classes rather than to the thousands of the rich. Nor need this be regretted. It is of the first importance that every man and woman should realise that the hospitals are theirs, ever ready to succour them in accident or disease, and dependent upon them for the means of continuing their beneficent work. But that is by no means all that needs to be realised. The cure of disease is a part only of the activities of the hospital—every year they become more and more important as centres of research. "It is even yet too little recognised by the public," says Sir Napier Burnett, "that the great and ever expanding science of preventive medicine owes its existence in large measure to the work carried out in hospital laboratories." From institutions intended for the cure of disease the hospitals have been evolved into schools of preventive medicine—that great science of the future. These activities are little more than a quarter of a century old, and it is probable that, provided the money is forthcoming, the next ten years will see a far greater advance in this direction than has been possible in the last five-and-twenty. This is one of the great works of the immediate future; another must necessarily be the extensions which have become urgently necessary during the last decade. These extensions will make a heavy demand upon, we will not say the purse of the charitable, because we must get rid of the idea of charity in relation to the hospitals, but upon the good will and understanding of those who desire them to reach the highest measure of efficiency as quickly as possible.

While the financial recovery of the hospitals is the outstanding fact of Sir Napier Burnett's report, it contains much else that is of real importance in regard to such matters as the presentation of statistics, the need for further laboratory accommodation, the position of cottage hospitals, and the food of patients. Hospital cuisine is a subject of very real importance which has hitherto received scant attention. It is notorious that the food both of patients and nurses is often plain to the point of repulsion. Even when its quality is above suspicion, which is not invariably the case, it is too often badly cooked and served in an unappetising way, while its sameness is exasperating. Monotonous and uninteresting food is in itself an offence against the laws of health, and there is a touch of the ironic in the fact that this offence should so systematically be committed in the hospitals. In some cases the medical staffs have insisted upon the appointment of Kitchen Committees, and we hope that in time such committees will be general. We are slowly learning that diet is more important than drugs, and that, in any case, it exercises a powerful influence not only over recovery in sickness but over the maintenance of health. Whether we are well or ill, "plain food" is best for all of us, but it must needs be well cooked and served in an appetising way. Better a dinner of savoury herbs than an ill-roasted ox, and a good hospital cook would be as powerful an allay of the medical staff as a well-trained nurse. That the nurse should be a highly-trained specialist and the cook a blundering amateur is a state of things which ought not to be endured with complacency.

